# Thermal Behavior Augments Heat Loss Following Low Intensity Exercise

**DOI:** 10.3390/ijerph17010020

**Published:** 2019-12-18

**Authors:** Nicole T. Vargas, Christopher L. Chapman, Blair D. Johnson, Rob Gathercole, Matthew N. Cramer, Zachary J. Schlader

**Affiliations:** 1 Center for Research and Education in Special Environments, Department of Exercise and Nutrition Sciences, University at Buffalo, Buffalo, NY 14214, USA; ntvargas@buffalo.edu (N.T.V.); cc338@buffalo.edu (C.L.C.); blairjoh@buffalo.edu (B.D.J.); 2 Lululemon Athletica Inc., Vancouver, BC V6J 1C7, Canada; rgathercole@lululemon.com; 3 Institute for Exercise and Environmental Medicine, Texas Health Presbyterian Hospital Dallas and University of Texas Southwestern Medical Center, Dallas, TX 75231, USA; matt.n.cramer@gmail.com; 4 Department of Kinesiology, School of Public Health, Indiana University, Bloomington, IN 47405, USA

**Keywords:** thermoregulatory behavior, thermoregulation, exercise recovery, voluntary cooling

## Abstract

We tested the hypothesis that thermal behavior alleviates thermal discomfort and accelerates core temperature recovery following low intensity exercise. *Methods:* In a 27 ± 0 °C, 48 ± 6% relative humidity environment, 12 healthy subjects (six females) completed 60 min of exercise followed by 90 min of seated recovery on two occasions. Subjects wore a suit top perfusing 34 ± 0 °C water during exercise. In the control trial, this water continually perfused throughout recovery. In the behavior trial, the upper body was maintained thermally comfortable by pressing a button to receive cool water (3 ± 2 °C) perfusing through the top for 2 min per button press. *Results:* Physiological variables (core temperature, *p* ≥ 0.18; mean skin temperature, *p* = 0.99; skin wettedness, *p* ≥ 0.09; forearm skin blood flow, *p* = 0.29 and local axilla sweat rate, *p* = 0.99) did not differ between trials during exercise. Following exercise, mean skin temperature decreased in the behavior trial in the first 10 min (by −0.5 ± 0.7 °C, *p* < 0.01) and upper body skin temperature was reduced until 70 min into recovery (by 1.8 ± 1.4 °C, *p* < 0.05). Core temperature recovered to pre-exercise levels 17 ± 31 min faster (*p* = 0.02) in the behavior trial. There were no differences in skin blood flow or local sweat rate between conditions during recovery (*p* ≥ 0.05). Whole-body thermal discomfort was reduced (by −0.4 ± 0.5 a.u.) in the behavior trial compared to the control trial within the first 20 min of recovery (*p* ≤ 0.02). Thermal behavior via upper body cooling resulted in augmented cumulative heat loss within the first 30 min of recovery (Behavior: 288 ± 92 kJ; Control: 160 ± 44 kJ, *p* = 0.02). *Conclusions:* Engaging in thermal behavior that results in large reductions in mean skin temperature following exercise accelerates the recovery of core temperature and alleviates thermal discomfort by promoting heat loss.

## 1. Introduction

Autonomic thermoregulatory responses during exercise recovery have been well described [1,2,3,4,5]. Even during relatively low intensity exercise, the post exercise period is characterized by paradoxically elevated core temperatures that occur as a consequence of autonomic thermoeffectors being rapidly withdrawn [1,6]. It follows that evaporative heat loss is attenuated, resulting in body heat storage above pre-exercise levels for up to an hour following cessation of exercise [1]. We recently identified that while autonomic heat loss effector responses are withdrawn, behavioral thermoeffector responses remain engaged following exercise [6,7]. The reason for this may be due to sustained elevations in core temperature, resulting in decreases in thermal comfort, which is defined as subjective indifference with the thermal environment [8]. Thermal discomfort (the reciprocal of thermal comfort) is the precursor to thermal behavior [9]. Core temperature is an important stimulus that contributes to thermal behavior during exercise recovery [6,10]. Therefore, it is possible that thermal behavior remains engaged following exercise in an effort to alleviate thermal discomfort and promote heat loss, which could facilitate a more rapid return of core temperature to pre-exercise levels [6]. However, this remains speculative given that previous studies only employed a local stimulus that provided an indication of both the magnitude and desire to behaviorally thermoregulate following exercise, but did not provide sufficient cooling to affect mean skin or core temperatures [6,11]. Therefore, while it seems obvious, the efficacy of thermal behavior following exercise has never been explored.

We recently identified that voluntarily cooling the skin of the upper body during exercise promotes dry heat loss and thermal comfort, despite attenuating sweat rate [12]. Given that sudomotor activity is rapidly withdrawn during exercise recovery, engaging in thermal behavior that promotes dry heat loss at this time, may accelerate the recovery of core temperature to pre-exercise levels. However, thermal behavior during recovery has never been formally examined. Notably, such an investigation may be applicable to both the lay public and athletic populations by identifying means of improving heat loss following physical activity or exercise. Moreover, populations that have thermoregulatory dysfunction (e.g., older adults, people with Multiple Sclerosis, etc.) may directly benefit from identifying ways to improve heat loss following exercise. Therefore, the present study tested the hypothesis that voluntarily cooling the skin of the upper body during recovery from low intensity exercise alleviates thermal discomfort and accelerates the restoration of core temperature occurring secondary to improvements in heat loss.

## 2. Materials and Methods

This study was approved by the Institutional Review Board at the University at Buffalo and conformed to the most recent standards set by the Declaration of Helsinki. Written consent was obtained from all subjects after they were fully informed of the experimental procedures and possible risks prior to completing in any study related activities.

### 2.1. Subjects

Twelve healthy young adults (6 females, age: 24 ± 3 years, height: 171 ± 10 cm, weight: 73 ± 14 kg, body surface area: 1.8 ± 0.2 m^2^, body fat: 16.4 ± 6.6%) participated in this study. All subjects were free from any known cardiovascular, metabolic, neurologic or psychological diseases. Female subjects were not pregnant, confirmed via a urine pregnancy test and were not breast-feeding, confirmed verbally. All females were self-reported to be normally menstruating and were tested within the first 10 days of their menstrual cycle, a period where progesterone and estrogen are at their lowest concentrations. Subjects visited the laboratory on three occasions. Visit one was the consent visit and visits two and three were experimental trials. Data from one of these experimental trials (i.e., the control trial) have been reported previously in a study that tested a different hypothesis [12]. The behavior trial in the current study was a completely separate trial from those presented previously in that subjects were restricted from behaving during exercise and only allowed to behave during recovery. As a result, this study tested a completely different hypothesis than our previous study by focusing on the thermal behavior responses following exercise when thermal behavior was not permitted during exercise. Upon arrival to the laboratory for the experimental trials, subjects refrained from caffeine, alcohol and exercise for at least 12 h and from food for 2 h and were instructed to arrive well hydrated.

### 2.2. Instrumentation and Measurements

Height and weight were measured with a stadiometer and scale (Satorius Corp. Bohemia, NY, USA). Body surface area was calculated accordingly [13]. Seven sites were measured for skinfold thickness at the chest, axilla, triceps, sub scapula, abdomen, suprailiac and thigh (Harpenden, Baty International, West Sussex, UK). Body density was calculated from the sum of skinfold measurements for males [14] and females [15], and used to estimate percent body fat [16]. Cognitive ability was measured using the Montreal Cognitive assessment [17] to ensure subjects were within a normal range. This was important due to the perceptual questionnaires and behavioral paradigm used in the study. Activity levels of all subjects were determined from the International Physical Activity Questionnaire [18]. Urine specific gravity was measured in duplicate using a refractometer (Atago USA, Inc., Bellvue, WA, USA) to ensure subjects were euhydrated prior to completing the experimental trials.

Beat to beat blood pressure was continually measured via the Penaz method (Finometer Pro, Finapres Medical Systems (FMS), Amsterdam, The Netherlands), which was confirmed via manual auscultation of the brachial artery. Heart rate was measured via a wireless transmitter (Polar, Kempele, Finland). Skin blood flow was measured continually on the dorsal surface of the left forearm under the water perfused top via integrated laser Doppler flowmetry (Periflux System 5010, Perimed, Stockholm, Sweden). Cutaneous vascular conductance was calculated as skin blood flow perfusion units divided by the mean arterial pressure.

Metabolic data were obtained via a mouthpiece with a one-way non-rebreathing valve at 10 and 20 min pre-exercise and every 30 min throughout exercise and recovery. Due to technical issues, minute ventilation was calculated from expired airflow measured via a heated pneumotachometer (Hans Rudolph, Inc., Shawnee, KS, USA, *n* = 7) or flow turbine (Vacumetrics, Inc., Ventura, CA, USA, *n* = 5). These data were continuously integrated over 1 min and corrected to standard temperature, pressure, dry (STPD). Whether expired airflow was measured using the pneumotachometer or flow turbine was kept constant within a subject. The fractions of expired oxygen and carbon dioxide (VacuMed, Ventura, CA, USA) were continuously measured from a 3 L mixing chamber. Oxygen uptake and carbon dioxide production were calculated using the Haldane Transformation. The rate of metabolic heat production was calculated from oxygen uptake and the respiratory exchange ratio (see Appendix A).

Core temperature was measured using a wireless telemetry pill (HQ Inc., Palmetto, FL, USA) that was ingested approximately 60 min prior to any experimental testing. One subject had contraindications to swallowing the core temperature pill. In this subject, rectal temperature was measured using a rectal thermistor (Mon-a-therm, Mallinckrodt Medical, Inc., St Louis, MO, USA) inserted 10 cm beyond the anal sphincter. Mean skin temperature was measured as the unweighted average of ten thermocouples attached to the left side of the body on the lower shin, posterior calf, posterior thigh, anterior thigh, abdomen, chest, supra-scapula, forearm, shoulder and on the middle of the forehead [19]. This unweighted average was chosen based on the recommendation that ten sites are most appropriate for studies examining thermal comfort [20].

Local sweat rate was measured by tightly securing a capsule that covered 3.9 cm^2^ of the skin 3–5 cm below the axilla, on the mid-axillary line (under the water perfused top, *n* = 12), and on the anterior thigh (outside of any clothing, *n* = 6 due to technical limitations). The axilla location was chosen because it is a location on the torso that is under the suit but is not prone to potential movement artifact that could have dislodged the sweat capsule from the skin. The thigh location was chosen to provide an index of local sweat rate outside of the water perfused suit. The capsule was tightly taped to the skin after applying it with double sided adhesive. Dry nitrogen was perfused through the capsule at a rate of 0.5 L/min, allowing for measurement of the water vapor from the skin exiting the gas capsules to be continuously measured by capacitance hygrometry (HMT130, Vaisala, Woburn, WA, USA). Local sweat rate was calculated by multiplying the humidity output by the flow rate of the dry nitrogen and dividing that value by the surface area of the capsule. Whole body sweat loss was estimated from the change in nude body weight pre- to post-trial and is reported in kilograms.

Relative humidity of the skin was measured via 8 hydrochron iButtons (Maxim Integrated Products Inc., San Jose, CA, USA) placed directly adjacent to a thermocouple at the forehead, chest, shoulder, forearm, supra-scapula, abdomen, anterior thigh and calf. At each location, the iButton was raised 6 mm off the skin using a custom-made capsule that allowed airflow to pass through. The distance of 6 mm was chosen because it ensured that the humidity sensor of the iButton would not become artificially supersaturated due to a droplet of sweat entering the hygrosensor [10,11]. Relative humidity from the iButtons and skin temperature from the adjacent thermocouple placed on each site were used to determine the water vapor pressure of the skin using standard calculations as previously reported [12,21]. Local skin wettedness was calculated according to the methods of Gagge [22] (See Appendix B). Whole body mean skin wettedness was calculated as the equally weighted average of all 8 local skin wettedness sites [11].

Thermal behavior was measured using a technique modified from those of Cabanac et al. [9,23] and currently employed by our laboratory [6,7,11,24]. However, in contrast to previous studies, thermal behavior in the present study involved voluntarily controlling the skin temperature of the torso and arms, a relatively large surface area of the body. Subjects controlled the temperature of their upper body by voluntarily perfusing cold water through a water perfused suit top (Med-Eng, Ottawa, ON, Canada) covering their arms and torso. Subjects were instructed to keep their body temperature thermally comfortable throughout exercise recovery but were not allowed to receive cooling during exercise. The water perfused suit top was continually perfused with thermoneutral water (34.0 ± 0.0 °C). Subjects were permitted to press a button any time they desired cold water (2.6 ± 2.3 °C) to perfuse the water perfused suit top. The temperature of the water baths was recorded in 5 min intervals. Pressing of the button initiated the turning of valves to allow cold water to run through the suit for 2 min. Following cooling, a mandatory 1 min wash out period was required, in which thermoneutral water again perfused through the suit top. This thermal behavioral approach (i.e., 2 min of cooling with a 1 min washout period) is analogous to operant (thermal) behavior paradigms used in non-human animal studies [25]. The advantage of this approach is that it ensures a continual drive to seek cooling. A compression top was placed over the water perfused top to ensure firm and even contact with the subject’s upper body. Perfusing cold water through the water perfused suit reduced upper body skin temperatures that were perceived within ~25 s. The unweighted average of upper body skin temperature (i.e., shoulder, forearm, chest, supra-scapula and abdomen) and the temperature of the effluent fluid exiting the water perfused suit top (i.e., water perfused suit top temperature) provided objective and continuous measures of thermal behavior [9,23]. 

Perceptual measures for the whole-body and upper body (i.e., torso and limbs) were taken every 10 min to the nearest 0.5 units using the following standard visual analogue scales: thermal sensation (1 = cold, 4 = neutral, 7 = hot [25]); thermal comfort (1 = comfortable, 4 = very uncomfortable [25]); and skin wettedness (+3 = very wet, +2 = wet, +1 = slightly wet, 0 = neutral, −1 = slightly dry, −2 = dry, −3 = very dry [21]) and sweating perception (0 = none, 10 = most ever [26]). These perceptual measures were chosen because thermal discomfort is the precursor to thermal behavior and thermal sensation provides information regarding the relative intensity of the temperature being sensed. Further, perceptions of skin wettedness have been considered an important factor in thermal behavior [21,27], which may also be informed by perceptions of sweating.

Finally, partitional calorimetry was used to estimate dry and evaporative heat loss both under and outside of the water perfused suit top. Using these data, dry and evaporative heat losses, and cumulative heat losses throughout recovery were estimated. Notably, this analysis was added post hoc based on the reported findings, as has previously been reported [12], to help inform decision making regarding conclusions. Details of the partitional calorimetry methods can be found in Appendix C. The same equations were employed as our previous study [12], but we calculated different specific correction factors from those reported previously.

### 2.3. Study Design and Experimental Protocols

At least 24 h prior to experimental testing, subjects reported to the laboratory and were familiarized with the water perfused top and the perceptual questionnaires. For the experimental trials, subjects arrived to the laboratory euhydrated, confirmed via urine specific gravity ≤ 1.020 (actual urine specific gravity—Control: 1.004 ± 0.006; Behavior: 1.004 ± 0.006), and having refrained from strenuous exercise, alcohol and caffeine for 12 h, and food for 2 h. The experimental trials were performed at the same time of day for each subject. During both trials, thermoneutral water perfused the suit top throughout 60 min of steady state exercise. Following exercise, a 90 min recovery period commenced in which thermoneutral water continued to perfuse through the suit top in both trials. During the behavior trial, subjects were free to behaviorally thermoregulate (i.e., receive ~2 °C water through the water perfused top) for 2 min at a time when they perceived themselves to be thermally uncomfortable. The control and behavior trials were separated by a minimum of 48 h. The control trial was always performed first so that subjects had a reference of the warmth and thermal discomfort generated by exercise in the most uncomfortable condition. All experimental testing was conducted during the summer months in Buffalo, NY (outside temperature on experimental days−Control: 19 ± 4 °C; Behavior: 20 ± 4 °C). Male subjects were shirtless under the water perfused top and females wore only a standard sports bra (energy bra, Lululemon Inc.,Vancouver, BC, Canada). Both males and females wore running shorts (men or women’s cut, Lululemon Inc.), and their own socks and athletic shoes. 

The experimental trials took place in a moderate thermal environment (Control: 27 ± 0 °C, 48 ± 11% relative humidity, Behavior: 27 ± 0 °C, 49 ± 6% relative humidity) with low air flow (≤ 0.1 m/s) to avoid lower leg exposure to air flow. Upon arrival at the laboratory, subjects ingested the wireless telemetry sensor and recorded their nude weight in a private room. Following ingestion of the pill and nude weight, subjects were not allowed to eat or drink anything until after the protocol was complete and a final nude body weight obtained. Subjects were then instrumented and sat on a mesh chair behind a standard upright cycle ergometer (Monark 828E, Vansbro, Sweden) for a 20 min baseline measurement period. Subjects remained in the recumbent position and began cycling on the ergometer for 60 min at a low intensity (i.e., approximately 70 W), followed by a 90 min recovery in the same recumbent position where they could employ thermal behavior in the behavior trial. A low intensity cycle protocol was chosen to help elucidate how behavioral and autonomic thermoeffectors interact during transitional periods following common exercise situations that may occur throughout the day (i.e., individuals who go for a walk at lunch time, or ride a bike to work). These types of protocols are also applicable to special populations who are commonly prescribed low intensity exercise. We have used similar protocols to study behavioral thermoregulation in the past [6,10,11] whereby a mild stimulus of an increase of ~0.5 °C core temperature has been shown to stimulate thermal behavior during, and following exercise, but these studies did not show the true effectiveness of thermal behavior. Subjects watched non-stimulating documentaries throughout the entire protocol.

### 2.4. Data and Statistical Analyses

Data were continuously recorded at 125 Hz and binned as 60 s averages every 10 min (Biopac MP160, Goleta, CA, USA). The time taken for core temperature to return to pre-exercise levels for each subject was identified using the minute at which core temperature returned to baseline within ±0.1 °C from the pre-exercise value. We analyzed core temperature data using this level of precision because the measurement of intestinal temperature is accurate to the nearest 0.1 °C [28]. In the event that core temperature did not return to pre-exercise values, a 90 min (i.e., the full duration of the recovery period) was used the analysis. Metabolic heat production during exercise, whole body sweat loss, cumulative heat loss and time for core temperature to return to pre-exercise levels were analyzed using a paired t-test. All other data were analyzed using a two-way (time × condition) repeated measures ANOVA. Data analyzed for the exercise recovery period include the pre-exercise time point, end-exercise time point, and every 10 min during recovery. When the ANOVA revealed a significant F test for interaction or main effect, a priori Sidak post hoc comparisons were made between trials and overtime compared to the 60 min end exercise time point. For all analyses, a priori statistical significance was set at *p* ≤ 0.05. Data are reported as mean ± SD. Actual *p*-values are reported for post hoc comparisons when there was a significant interaction or main effect, or for the overall ANOVA when no interaction or main effect was found. 

## 3. Results

### 3.1. Exercise Stimulus

The average metabolic heat production (Control: 209 ± 22 W⋅m^2^; Behavior: 212 ± 32 W⋅m^2^, *p* = 0.75), average absolute external workload (Control: 70 ± 1 W; Behavior: 70 ± 2 W, *p* = 0.95), heart rate (Control – 30 min: 110 ± 14 bpm, 60 min: 111 ± 13 bpm; Behavior – 30 min: 110 ± 12 bpm, 60 min:111 ± 13 bpm, *p* = 0.88) and mean arterial pressure (Control – 30 min: 85 ± 13 mmHg, 60 min: 84 ± 13 mmHg; Behavior – 30 min: 83 ± 11 mmHg, 60 min: 82 ± 11 mmHg, *p* = 0.71) did not differ between trials.

### 3.2. Exercise Recovery

#### 3.2.1. Body Temperatures

**Figure 1:**
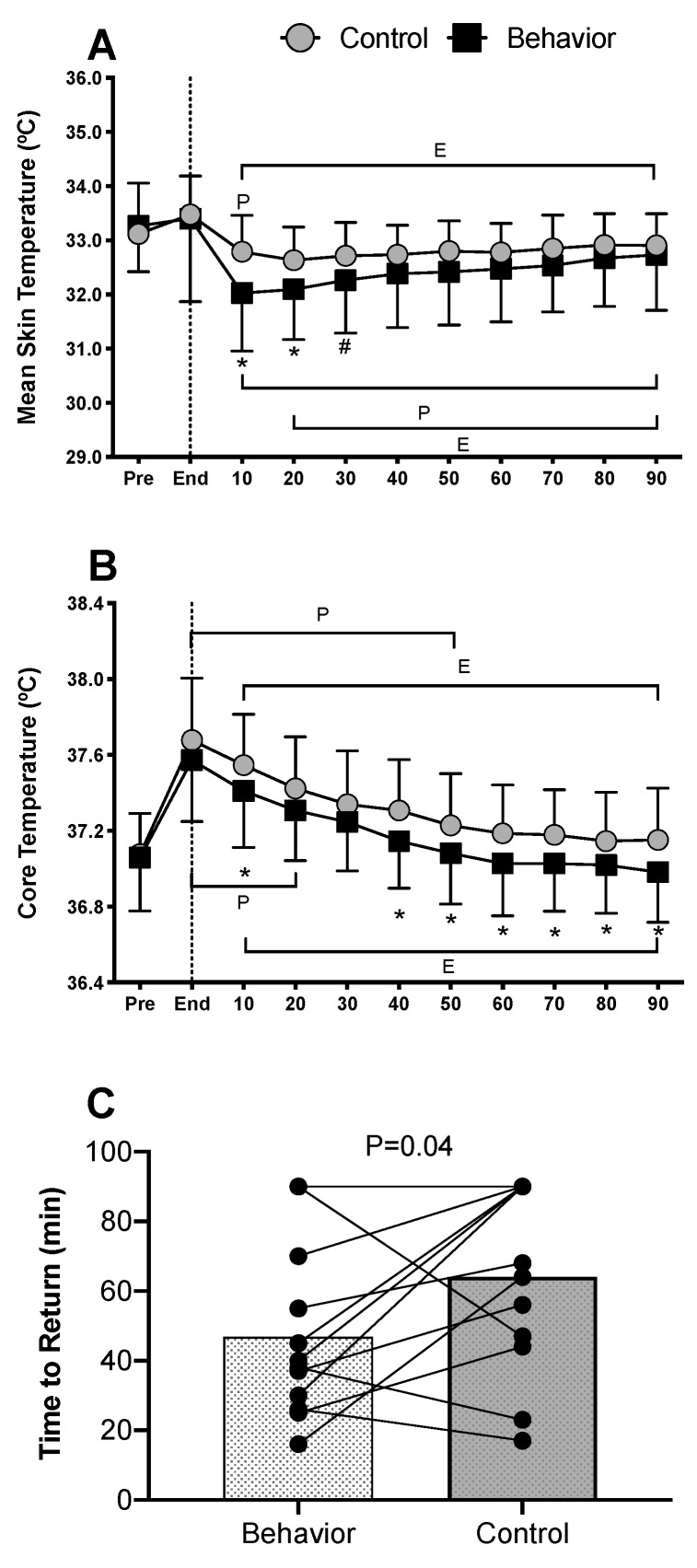
Body temperatures (*n* = 12, mean ± SD). (**A**) Mean skin temperature, (**B**) core temperature and (**C**) time for core temperature to return to pre-exercise levels, during 90 min of recovery from low intensity cycling exercise. ^P^ Different from pre-exercise (*p* < 0.03); ^E^ Different from end-exercise (*p* < 0.01); * Behavior different from control (*p* ≤ 0.04); ^#^ Behavior different from control (*p* = 0.06).

Mean skin temperature was not different between conditions at pre- (*p* = 0.99) or end-exercise (*p* = 0.99) and did not increase from baseline during exercise in either condition (*p* ≥ 0.06). Mean skin temperature decreased from the end-exercise time point in both conditions within the first 10 min of recovery (*p* < 0.01), was reduced to a greater extent in the behavior trial compared to the control trial within the first 10 min (by −0.8 ± 1.0 °C) and remained lower through 20 min of recovery (*p* ≤ 0.01) (Figure 1A). Core temperature was not different between control and behavior trials at pre- (*p* = 0.99), during, or end-exercise (*p* = 0.18), but increased within the first 20 min of exercise in both conditions (*p* < 0.01). Core temperature began to decrease within the first 10 min of recovery in both trials (*p* ≤ 0.02) and returned to pre-exercise levels within 40 min of recovery in the behavior trial (*p* = 0.38), but not until 60 min in the control trial (*p* = 0.12). Core temperature was different in the behavior compared to control trial at 10 min, and 40–90 min during recovery (*p* < 0.05) (Figure 1B). Notably, core temperature returned to pre-exercise levels quicker in the behavior compared to control trial (47 ± 25 vs. 64 ± 27 min, *p* = 0.04) (Figure 1C).

#### 3.2.2. Skin Wettedness and Skin Absolute Humidity

**Figure 2:**
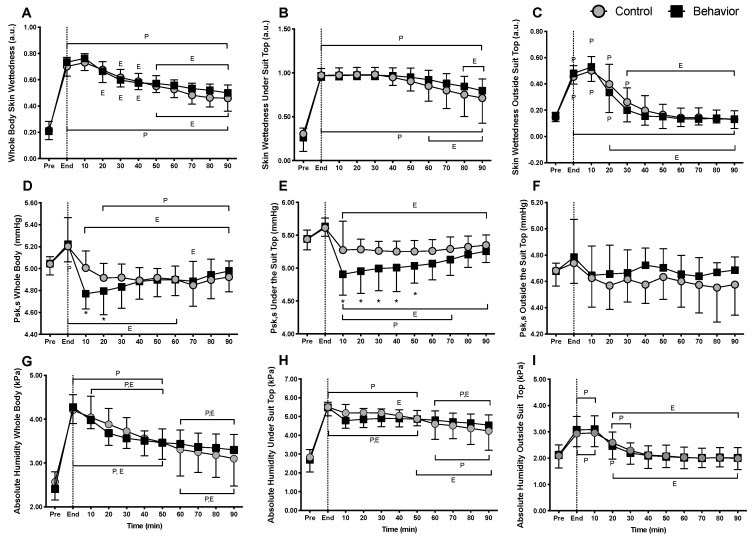
Skin wettedness, absolute skin humidity, and the saturated vapor pressure at the skin (P_sk,s_) (*n* = 12, mean ± SD). (**A**) Whole body skin wettedness, (**B**) under suit top skin wettedness, (**C**) outside suit top skin wettedness, (**D**) whole body absolute humidity, (**E**) under suit top absolute humidity, (**F**) outside suit top absolute humidity, (**G**) whole body P_sk,s_, (**H**) under suit top P_sk,s_, (**I**) outside the suit top P_sk,s_, during 90 min of recovery from low intensity cycling exercise. ^P^ Different from pre-exercise (*p* ≤ 0.04); ^E^ Different from end-exercise (*p* ≤ 0.05); * Behavior different from control (*p* ≤ 0.05).

Skin wettedness for the whole body (*p* ≥ 0.19) (Figure 2A), under the suit top (*p* ≥ 0.99) (Figure 2B), and outside the suit top (*p* ≥ 0.09) (Figure 2C) did not differ between trials at any time. Whole body absolute humidity was lower in the behavior trial versus the control trial at 20 min into recovery (*p* = 0.05) (Figure 2D). Absolute humidity under the suit top was lower in the behavior trial versus the control trial at 10 min into recovery (*p* = 0.04) (Figure 2E), while absolute humidity outside the suit top did not differ between the behavior and control trials (*p* ≥ 0.44) (Figure 2F). Whole body saturated skin absolute humidity (P_sk,s_) did not differ between trials (*p* ≥ 0.32) (Figure 2G). P_sk,s_ under the suit was lower in the behavior trial at 10 and 20 min into recovery (*p* < 0.01) (Figure 2H), but P_sk,s_ outside the suit did not differ between trials (*p* ≥ 0.21) (Figure 2I).

#### 3.2.3. Thermoeffector Responses

**Figure 3:**
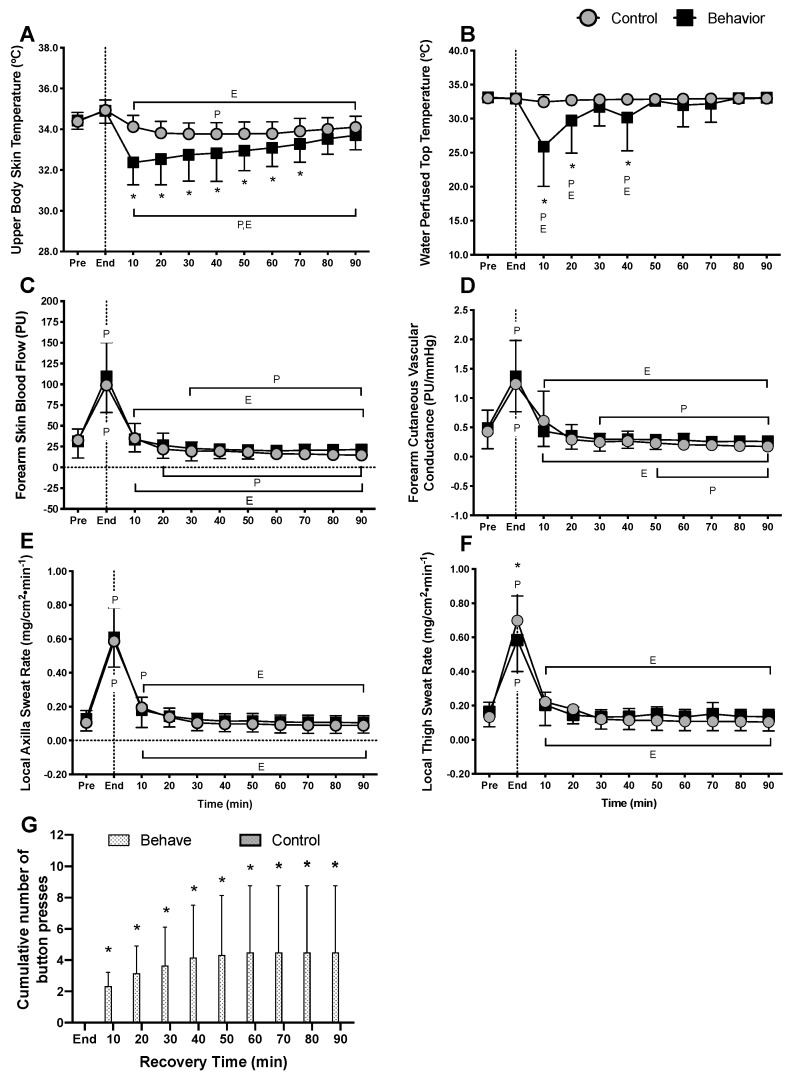
Thermoeffector responses (*n* = 12, mean ± SD). (**A**) Upper body skin temperature; (**B**) Water perfused top temperature; (**C**) Forearm skin blood flow; (**D**) Forearm cutaneous vascular conductance; (**E**) Local axilla sweat rate; (**F**) Local thigh sweat rate (*n* = 6); (**G**) Cumulative number of button presses, during 90 min of recovery from low intensity cycling exercise. ^P^ Different from pre-exercise (*p* ≤ 0.01); ^E^ Different from 60 min (end-exercise) (*p* < 0.02); * Behavior different from control (*p* ≤ 0.05).

The cumulative number of button presses allowed us to quantify when and how often subjects initiated thermal behavior during exercise recovery (Figure 3G). Cumulative button presses increased in the behavior trial 10 min into the recovery period and remained elevated throughout (*p* < 0.01), but after 40 min of recovery, subjects did not seek further cooling (Figure 3G). Upper body skin temperature and water perfused top temperature provided direct measures of thermal behavior. Both upper body skin and water perfused top temperatures were not different between conditions at pre- (*p* = 0.99), during (*p* = 0.99) or end-exercise (*p* = 0.99), but they increased during exercise (*p* < 0.01). Upper body skin temperature decreased from pre-exercise in the behavior trial from 10 min into recovery and onwards (*p* < 0.01). In both trials, upper body skin temperature decreased from end-exercise within the first 10 min during recovery (*p* < 0.01), however, upper body skin temperature was reduced to a greater extent in the behavior trial compared to control from 10 to 70 min into recovery (*p* ≤ 0.05) (Figure 3A). During recovery from exercise, water perfused top temperature was lower in the behavior trial compared to control trial at 10, 20 (*p* < 0.01) and 40 min of recovery (*p* = 0.04) (Figure 3B). Forearm skin blood flow (*p* = 0.29) (Figure 3C) and forearm cutaneous vascular conductance (*p* = 0.14) (Figure 3D) were not different at the pre- or end-exercise timepoints, or throughout recovery between trials. Axilla sweat rate was not different between conditions at any time point (*p* ≥ 0.97) but increased during exercise from pre-exercise levels (*p* < 0.01) (Figure 3E). Thigh sweat rate was higher at end-exercise in the behavior trial (*p* < 0.01) but did not differ between trials during recovery (*p* ≥ 0.83) (Figure 3F). Surprisingly, thigh sweat rate was higher at end-exercise in the control trial, despite exercise intensity being identical (*p* < 0.01). This unexpected finding may simply be a consequence of the low sample size for thigh sweat rate. Thigh sweat rate did not differ between trials during recovery (*p* ≥ 0.83) (Figure 3F). Whole body sweat losses between pre- and post-exercise were not different between trials (Control: 0.63 ± 0.20; Behavior: 0.59 ± 0.10, *p* = 0.24). 

#### 3.2.4. Perceptual Responses

**Figure 4:**
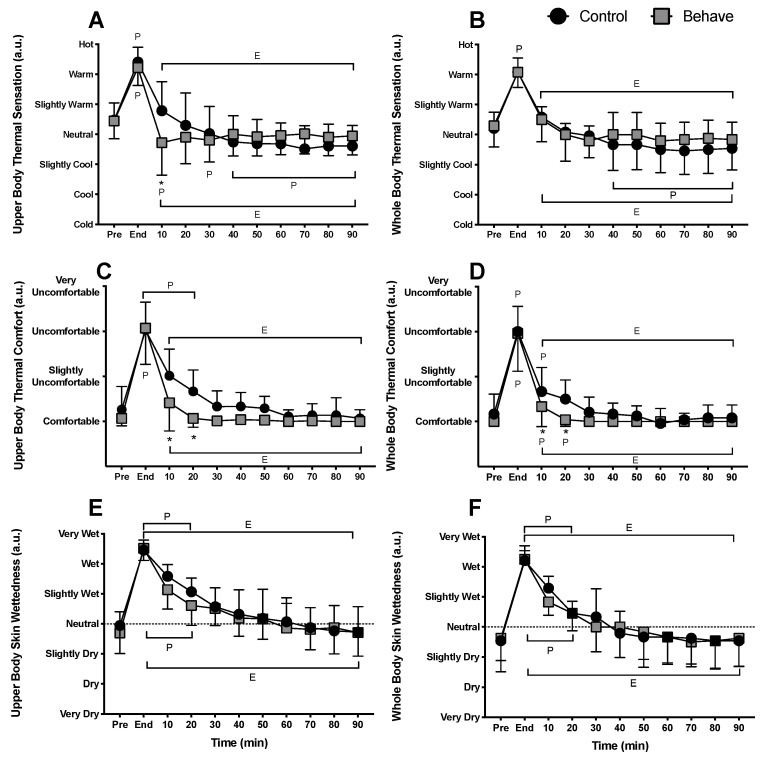
Perceptual responses (*n* = 12, mean ± SD) of (**A**) upper body thermal sensation, (**B**) whole body thermal sensation, (**C**) upper body thermal discomfort, (**D**) whole body thermal discomfort, (**E**) Upper body skin wettedness and (**F**) whole body skin wettedness during recovery from low intensity exercise. ^P^ Different to pre-exercise (*p* ≤ 0.05); ^E^ Different to end-exercise (*p* < 0.01); * Behavior different from control (*p* ≤ 0.03).

At pre-exercise in both conditions, subjects perceived their upper and whole body between neutral and slightly warm (*p* = 0.99). Following exercise, upper and whole-body perceptions were warm in both the control and behavior trials (*p* = 0.99). At 10 min into recovery during the behavior trial, upper body thermal sensation was cooler due to voluntary behavior compared to the control trial (*p* < 0.01). The perception of warmth in the upper body was reduced within the first 10 min into recovery in both trials compared to end-exercise, but to a greater extent in the behavior trial (*p* < 0.01) (Figure 4A). The perception of warmth in the whole body was not different between conditions at any time point (*p* ≥ 0.35) but was elevated during exercise compared to pre-exercise and decreased within the first 10 min into recovery compared to end-exercise (*p* < 0.01) (Figure 4B). 

Prior to exercise, there were no differences in upper and whole-body thermal comfort between conditions (*p* ≥ 0.75). Likewise, at the end of exercise, subjects perceived their upper and whole body to be thermally uncomfortable in both trials (*p* ≥ 0.99), however thermal discomfort in both the upper and whole body was completely alleviated (i.e., returned to pre-exercise levels) within the first 20 min of recovery in the behavior trial (*p* ≤ 0.03), while subjects were still slightly thermally uncomfortable in both the upper and whole body in the control trial until 30 min of recovery (*p* ≤ 0.03). There were no differences in upper or whole-body thermal discomfort after 30 min of recovery between trials (*p* ≥ 0.18) (Figure 4C,D).

Perceptions of upper and whole-body skin wettedness were not different between conditions at pre-exercise (*p* ≥ 0.87), end-exercise (*p* ≥ 0.47), or throughout recovery (*p* ≥ 0.07). The upper and whole body was perceived to have increased wettedness at end-exercise compared to pre-exercise (*p* < 0.01). Whole and upper body perceptions of skin wettedness remained elevated through 30 min recovery in the control (*p* < 0.01), while whole body perceptions of skin wettedness remained elevated through 20 min and upper body skin wettedness through 30 min of recovery for the behavior trial (*p* < 0.01). Both perceptions of upper and whole-body skin wettedness were reduced within 10 min of recovery in the control and behavior trials and continued to fall throughout the remainder (*p* < 0.01) (Figure 4E,F).

#### 3.2.5. Partitional Calorimetry

**Figure 5:**
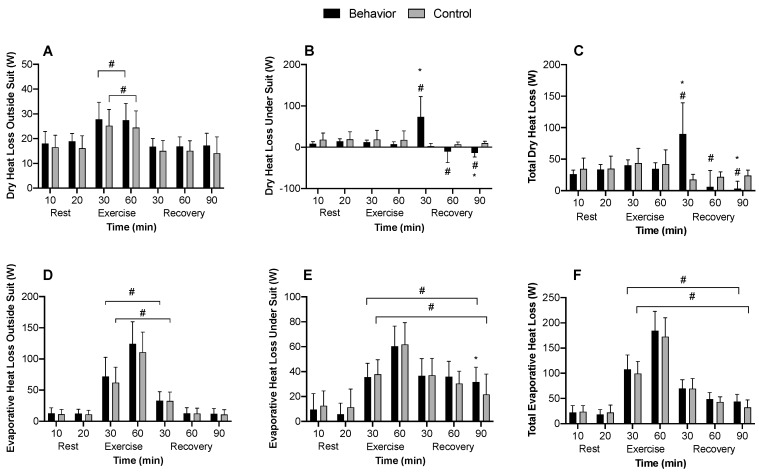
Dry and evaporative heat loss (*n* = 12, mean ± SD). (**A**) Dry heat loss outside the suit top, (**B**) dry heat loss under the suit top, (**C**) total dry heat loss, (**D**) evaporative heat loss outside the suit top, (**E**) evaporative heat loss under the suit top, (**F**) total evaporative heat loss during 90 min of recovery from low intensity cycling exercise. ^#^ Different from 60 min (end-exercise) (*p* < 0.01); * Behavior different from control (*p* ≤ 0.05).

**Figure 6:**
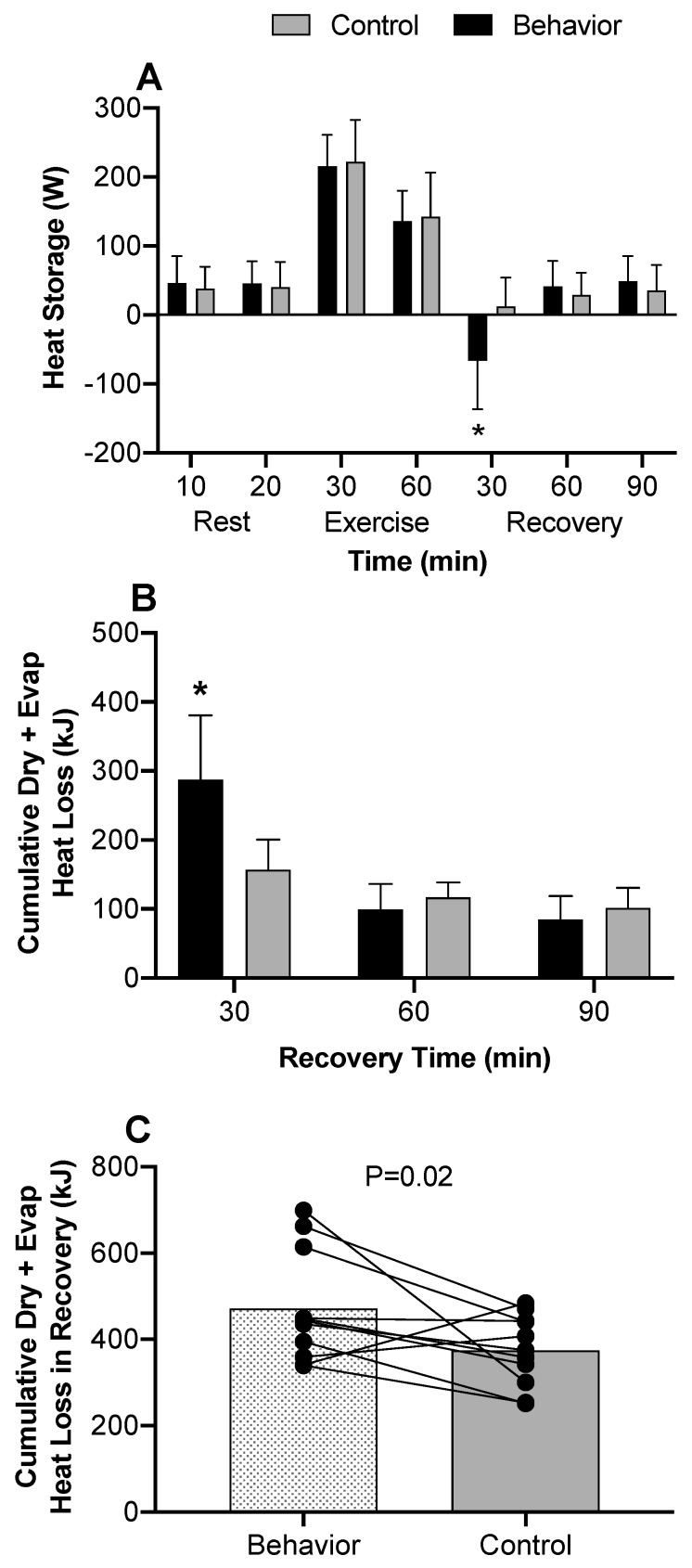
(**A**) Heat storage throughout exercise and recovery, (**B**) cumulative dry and evaporative heat loss throughout recovery and the sum of (**C**) cumulative dry and evaporative heat loss during 90 min recovery from low intensity exercise, (*n* = 12, mean ± SD). ^#^ Different from 60 min (end-exercise) (*p* < 0.01); * Behavior different from control (*p* ≤ 0.02).

Dry heat loss outside of the suit top was not different between trials but increased during exercise in both trials (*p* < 0.01) (Figure 5A). Dry heat loss under the suit top was elevated during the first 30 min of recovery in the behavior compared to control trial (*p* < 0.01). However, there was dry heat gain at 90 min into recovery in the behavior compared to control trial due to lower skin temperatures gaining heat from the water perfused top (*p* < 0.01) (Figure 5B). Overall, total dry heat loss was greater at 30 min in behavior compared to control (*p* < 0.01) but was less at 90 min compared to control (*p* = 0.03) (Figure 5C). Evaporative heat loss outside of the suit top also did not differ between trials during exercise (*p* ≥ 0.51) but increased and remained elevated at 30 min into recovery for both conditions (*p* < 0.01) (Figure 5D). Evaporative heat loss under the suit top was greater only at 90 min of recovery in the behavior compared to control trial (*p* = 0.01). Otherwise, both conditions had greater evaporative heat loss throughout exercise and recovery compared to baseline (*p* < 0.01) (Figure 5E). Total evaporative heat loss was not different between behavior or control trials at any time point (*p* ≥ 0.45), but increased in both conditions during exercise and remained elevated throughout recovery (*p* < 0.01), except at 90 min in the control trial, where it was not different to pre-exercise levels (*p* = 0.25) (Figure 5F). At 30 min recovery, total heat storage was negative (Figure 6A), and cumulative heat loss greater (Figure 6B), indicating augmented heat loss in the behavior compared to control trial (*p* < 0.01). All other time points saw similar heat storage (Figure 6A) which increased during exercise in both trials (*p* < 0.01). There was greater cumulative dry and evaporative heat loss in the first 30 min of recovery (*p* < 0.01) (Figure 6B), but no differences in the 60 and 90 min recovery time points (*p* ≥ 0.46). Total (i.e., sum of) cumulative dry and evaporative heat loss was greater in the behavior trial compared to the control trial during recovery (*p* = 0.02) (Figure 6C).

## 4. Discussion

The present study tested the hypotheses that during recovery from low intensity exercise, voluntarily cooling a large surface area of the body would alleviate thermal discomfort and accelerate the recovery of core temperature. The results indicate that thermal behavior improved thermal comfort for up to 30 min following low intensity exercise (Figure 4D) and accelerated the return of core temperature to pre-exercise levels (Figure 1C). These results are further supported by the partitional calorimetry data, which confirmed a greater heat loss within the first 30 min of recovery from exercise (Figure 5B,C), the point at which thermal behavior was most engaged (Figure 3B), and greater overall cumulative heat loss during recovery (Figure 6C).

It has previously been shown that with elevations in core temperature, skin temperatures that favor the return of core temperature to thermoneutral levels are considered comfortable, while those that exacerbate core temperature deviation from thermoneutral are uncomfortable [23,29,30]. In the present study, perceptions of whole body and upper body thermal discomfort were restored to comfortable levels in the behavior trial within the first 10 min of ceasing exercise, while they remained elevated in the control trial until 30 min (Figure 4C,D). The rapid restoration of thermal comfort in the behavior trial was likely initially due to increased cold-receptor activation and/or attenuated warm-receptor activation in the skin of the upper body due to subjects voluntarily cooling their skin temperature by up to −1.4 ± 0.8 °C. In contrast, the eventual return of thermal comfort in the control trial was more than likely due to the gradual withdrawal of warm-receptor activation as internal body temperature decreased without any active cooling that reduced skin temperature.

Following low intensity exercise in moderate environmental conditions (i.e., ~26 °C, 40% relative humidity), core and skin temperatures and skin wettedness contribute to thermal behavioral responses [6,10,11]. However, studies that identified these contributing factors to thermal behavior during exercise used local cooling models that did not allow for voluntary changes in heat exchange capable of modifying the physiological status of the body (i.e., [6,11]). In the present study, behavioral thermoregulation resulted in the modification of skin temperature of the full torso and arms, equating to about 54% body surface area. Notably, voluntarily cooling the skin following exercise increased dry heat loss, which expedited the recovery of core temperature by 17 ± 31 min compared to when voluntary behavior was not allowed (Figure 1C). It has previously been shown that following exercise, sudomotor and cutaneous vasodilation rapidly decrease [6,7], likely owing to reductions in blood pressure and subsequent baroreceptor unloading [2,31]. In the present study, mean arterial pressure and local sweat rates were reduced following end-exercise to a similar extent in both trials. Reductions in evaporative heat loss as a consequence of sudomotor withdrawal following exercise can result in elevated core temperatures and residual body heat storage above pre-exercise levels well into recovery [4]. It follows, therefore, that voluntarily cooling the skin may be beneficial in helping to accelerate the return of core temperature to pre-exercise levels. However, whether thermal behavior is engaged specifically to reduce core temperature or that thermal behavior occurs in an effort to alleviate thermal discomfort remains unknown. Nevertheless, to further understand interactions between reductions in core temperature and body heat loss due to behavioral thermoregulation, we employed partitional calorimetry as a post hoc analysis. The results indicate that following exercise, when subjects behaviorally thermoregulated and voluntarily reduced their skin temperature, this resulted in greater dry heat loss compared to the control trial (Figure 5B,C). Further to this, cumulative heat loss for the initial 30 min (Figure 6B) and overall 90 min recovery period was greater in the behavior trial (Figure 6C). These findings support that core temperature recovered quicker following exercise when thermal behavior was employed compared to when it was not, largely due to augmented dry heat loss.

Thermal behavior during recovery from exercise has been shown to be largely driven by skin wettedness, with core and mean skin temperature playing a lesser role [11]. Skin wettedness is dependent on the temperature of the skin and the water vapor pressure gradient between the skin and the air, relative to when the skin is fully saturated with water (i.e., P_sk,s_) [22]. In the present study, skin wettedness did not differ between the behavior and control trials (Figure 2A–C). This is likely explained by proportional reductions in skin absolute humidity (Figure 2D,E) and P_sk,s_ (Figure 2G,H) occurring secondary to lower skin temperatures. Given the important role for skin wettedness in thermal behavior during and following exercise [11], these findings are somewhat surprising, as it was expected that thermal behavior would reduce skin wettedness. However, these findings do support our previous work, which suggests that absolute skin humidity may be a more important factor that skin wettedness in contributing to decisions to behaviorally thermoregulate [12]. That said, it is possible that because the overall goal of thermal behavior is to aid in the regulation of body temperature, it may not be necessary for thermal behavior during exercise recovery to directly reduce skin wettedness. Rather, it may be that increased skin wettedness simply sensitizes the thermal behavioral response to seek cooling. Moreover, it has been suggested that the perception of skin wettedness, and not actual skin wettedness/humidity, may contribute to thermal discomfort and thermal behavior [32]. Skin wettedness is perceived by both mechanical and thermal inputs [27]. It is likely that, in the present model, mechanical inputs were perceived more readily given that skin cooling did not augment skin wettedness perception throughout exercise or recovery. This is in line with our observations of actual skin wettedness but raises the question as to whether humans are able to perceive skin wettedness or absolute humidity.

### 4.1. Considerations

Findings from the present study are applicable only to the environmental conditions and exercise protocols employed herein. It is important to further investigate if these observations would hold true during recovery from exercise in more stressfully warm or cool environmental conditions. For instance, it remains unknown whether voluntary cooling is effective at restoring thermal comfort and elevations in core temperature following higher intensity exercise given evidence that thermal behavior is more rapidly withdrawn during recovery from high compared to low intensity exercise [10]. Hence, we speculate that the results in the present study would likely differ following higher intensity exercise. In addition to this limitation, there remains some uncertainty surrounding thermal behavior and the menstrual cycle with some research suggesting that thermal behavior does not differ throughout the menstrual cycle [33], while others report differences [34,35]. For this reason, we limited our female testing to the follicular phase of the menstrual cycle. However, we did not confirm menstrual cycle phase via hormonal analyses. A further consideration is that we did not intend to calculate partitional calorimetry a priori. As such, there are specific correction factors that were determined post hoc. There are some limitations to this approach. For example, we do not know how much of the water perfused suit top was wet from sweating in each individual, and therefore cannot account for impeded or accelerated heat transfer via this medium. Nevertheless, we employed a cross-over design with each subject serving as their own control. Therefore, any errors are likely systematic and uniform across all calculations. It could be argued that our study may have been improved if the two trials were randomized. However, that the control trial was always conducted first was by design to ensure that all participants experienced the experimental conditions prior to the behavior trial. This was deemed necessary given that exercising in a water perfused suit with 34 °C water perfusing it is not a normal situation. Admittedly, this could have been averted had all participants undergone a familiarization trial before being randomly assigned to the two trials. However, this was considered unnecessary given that the thermoregulatory responses to the control trial were autonomically controlled, thus an order effect would be unlikely. Finally, while our subject group was represented by six females and six males, we were under powered to statistically look at sex differences. However, it would be interesting to investigate sex differences in this model, considering early research from our laboratory did reveal sex differences during exercise recovery when a local cooling model was employed [7]. Finally, we note that these data are not likely of clinical significance considering the small (i.e., 0.1–0.2 °C) differences in core temperature between trials. Nevertheless, there is a possibility that these data are of physiological significance, particularly if core temperature increases to a greater extent with exercise (e.g., as would occur if exercise intensity were higher). Furthermore, that core temperature returned to pre-exercise levels nearly 20 min quicker when participants were allowed to engage in cooling, emphasizes the finding that heat loss can be augmented following exercise to help the recovery of core temperature. These findings may be applicable to a large portion of the exercising population.

### 4.2. Perspectives

The data presented herein support common perceptions that employing thermal behavior (i.e., fanning, taking a cold shower, or donning a cool vest) during recovery from exercise may be beneficial for alleviating thermal discomfort and for accelerating the recovery of core temperature. Importantly, the post-exercise time frame is characterized by residual heat storage that occurs because of autonomic thermoeffector withdrawal. In contrast to our earlier study in which thermal behavior was not shown to improve heat loss, but rather supplemented dry for evaporative heat loss [12], these data highlight the benefits of employing thermal behavior during recovery from exercise to help manage residual heat storage within the recovery period. Thus, strategies for voluntarily cooling the skin to promote heat loss are beneficial for both augmenting the recovery of core temperature and restoring thermal comfort [36,37]. These findings have broad applications to the population ranging from recreationally active individuals to the elite athlete [38], and those with altered thermoregulatory ability [39,40,41] looking to promote heat loss and improve thermal discomfort post-exercise. Moreover, these findings may be particularly beneficial for athletes and spectators at international competitions expected to be hot and/or humid (e.g., the 2020 Tokyo Olympics) where cooling strategies to promote heat loss will be of utmost importance in order to mitigate the risk of heat illness [42] and alleviate thermal discomfort. 

## 5. Conclusions

Following low intensity exercise, thermal behavior that reduces skin temperature on a large body surface area is an effective way to voluntarily promote heat loss, accelerate the return of core temperature to pre-exercise levels, and alleviate thermal discomfort. 

## Appendix A. Metabolic Heat Production Calculations

Estimates of metabolic heat production (H_prod_) were calculated via partitional calorimetry in watts (W), using the following equation:

(1) $$H_{prod}=M-Wk\;\left(W\right)$$

where M is the metabolic energy expenditure, calculated in Equation (2) below and Wk is the external workload calculated in Equation (3) below [43]:

(2) $$M=VO_{2}\times \frac{\left[\left(\frac{RER-0.7}{0.3}\right)\times 21.13\right)+\left(\left(\frac{1.0-RER}{0.3}\right)\times 19.62\right)}{60}\;\times 1000\;\left(W\right)$$

where RER is the respiratory exchange ratio. And 21.13 represents the caloric equivalent per liter oxygen for the oxidation of carbohydrate, and 19.62 that for oxidation of fat [43].

(3) Wk=rpm×kp (W)

where rpm is the cadence and kp is the kilopond resistance applied to the ergometer.

## Appendix B. Skin Wettedness Calculations

Local skin wettedness was calculated at each site as the ratio between the evaporative heat flux gradient between the humidity at the skin and in the air, and the maximal evaporative heat flux gradient for a totally wet skin [21,22]:

(4) $$W_{local}=\frac{P_{sk}-P_{a}}{P_{sk,s}-P_{a}}\;\left(a.u.\right)$$

where P_sk_ is the measured water vapor pressure at the skin and P_a_ is the partial pressure of water in the atmosphere measured in kilopascals (kPa), calculated as:

(5) $$P_{sk}=\left(\frac{Rh_{sk}}{100}\right)\times P_{sk,s}\;\left(\mathrm{kPa}\right)$$

where Rh_sk_ is the relative humidity measured from the respective iButton, placed 6mm off the skin surface [11] and P_sk,s_ is the saturated vapor pressure at the skin. P_a_, can be calculated from Equation (5) by substituting Rh_a_ as the relative humidity measured within the environmental chamber, and P_a,s_ as the saturated water vapor pressure in the air. P_sk,s_ can be calculated as:

(6) $$P_{sk,s}=0.1\exp\left(18.956-\frac{4030.18}{T_{sk}+235}\right)\;\left(\mathrm{kPa}\right)$$

and P_a,s_ can be calculated using Equation (6), substituting T_a_ for T_sk_ [43]. Whole body skin wettedness was calculated as the unweighted average of all 8 sites.

## Appendix C. Rate of Heat Storage Calculations

To further understand heat loss mechanisms in the recovery period, post hoc analyses were performed to determine heat storage in each trial, and whether or not cumulative dry and evaporative heat losses differed between trials. The rate of heat Storage (S) was calculated every 15 min during baseline and every 30 min during exercise. Heat storage was calculated as:

(7) $$S=H_{prod}-H_{dry}-H_{evap}-H_{res}\;\left(W\right)$$

where H_dry_ represents the sum of dry heat losses from radiation and convection, H_evap_ represents heat loss from evaporation and H_res_ represents heat loss from respiration. The rate of heat loss from conduction was considered negligible and thus was eliminated from the equation [44]. Calculations for H_dry_ and H_evap_ were performed for areas outside and under the suit and summed together to calculate total H_dry_ and H_evap_ losses. H_dry_ from convection and radiation outside the suit were calculated as:

(8) $${\left(C_{skin}+R_{skin}\right)}_{outside}=\left(T_{sk}-T_{a}\right)\times \left(h_{r}+h_{c}\right)\times BSA\;\left(W\right)$$

where T_sk_ is the mean skin temperature of the legs and head, T_a_ is the ambient temperature of the environmental chamber, h_r_ and h_c_ are the estimated radiative and convective heat transfer coefficients, respectively and were calculated as previously described by Jay and Kenny [45]. BSA was the body surface area not influenced by the water perfused suit top, estimated to be 60% [46]. The ambient temperature was used as the air and radiant temperatures were assumed to be equivalent [44]. H_dry_ from convection and radiation under the water perfused suit top was adapted from previous equations and calculated as:

(9) $${\left(C_{skin}+R_{skin}\right)}_{under}=\left(T_{bath}{}_{out}-T_{bath}{}_{in}\right)\times C_{p}{}_{fluid}\times M_{fluid}\;\left(W\right)$$

where T_bath_out_ was always 34 °C for the control trial, and calculated for each subject based on the number of behaviors performed during each 30 min block throughout the cycling protocol during the behavior trial. T_bath_in_ was directly measured as the temperature of the water immediately after perfusing the top. The C_p_fluid_ is the specific capacity of water, (4.184 J⋅g^−1^⋅°C^−1^) and M_fluid_ is total mass of water perfusing the top in a 30 min period (18 L total volume). A correction factor was applied to account for the dry heat lost (in the control trial) or gained (in the behavior trial) from the environment to the suit alone. To determine the correction factor, the suit was placed on a manikin equilibrated to an ambient temperature of 27 °C and 40% relative humidity to simulate average temperature and humidity in the experimental trials. The average T_bath_out_ temperature perfusing the suit for all subjects (Control: 34 °C; Behavior: 31.0 °C) was perfused through the suit top for 60 min. The temperature of the water immediately after perfusing through the top (T_bath_in_) was also measured. The correction factor was calculated individually for the control and behavior trials as:

(10) $$H_{dry}Correction\;Factor=\left(T_{bath}{}_{out}-T_{bath}{}_{in}\right)\times C_{P}{}_{fluid}\times M_{fluid}\;\left(W\right)$$

for each 30 min period and averaged together. These factors were 56.8 W of heat lost from the suit to the environment when 34 °C water perfused the suit top, and 21.2 W of heat gained to the suit from the environment when 31.0 °C water perfused through the top. The final corrected H_dry_ under the suit was calculated as:

(11) $${\left(C_{skin}+R_{skin}\right)}_{suit}=\left[{\left(C_{skin}+R_{skin}\right)}_{uncorrected}-{\left(C_{skin}+R_{skin}\right)}_{corrected}\right]\times \;BSA\;(W)$$

where BSA is the area directly in contact with the water perfused suit top, estimated to be 40% [46]. All data were calculated in W and then converted to kJ. Dry heat loss from outside the suit and under the suit were summed together as a measure of total dry heat loss.

Heat loss from evaporation was also individually calculated for areas outside and under clothing. H_evap_ from outside the suit was calculated as:

(12) $$E_{outside}=\;h_{e}\times \left(P_{sk}-P_{a}\right)\times BSA\;\left(W\right)$$

where h_e_ is the product of the convective heat transfer coefficient and the Lewis Relation coefficient (16.5 K⋅kPa^−1^), and accounting for the barometric pressure (P_b,_ (mmHg)) and calculated as:

(13) $$h_{e}=16.5\;\times h_{c}\times \;\left(\frac{760}{P_{b}}\right)\;(W\cdot m^{-2}\cdot {\mathrm{kPa}}^{-1})$$

P_sk_ is the absolute partial pressure of water on the skin calculated from the relative humidity measured at the skin via each local iButton (Equation (5) above), P_a_ is the absolute partial pressure of water in the air calculated from relative humidity and temperature (Equation (5) above, substituting ambient relative humidity and temperature), and BSA is the relative body surface area inside or outside the water perfused top. P_sk_ was calculated using iButtons locally placed at the, calf, anterior thigh and forehead.

To determine evaporation from under the water perfused suit top, iButtons locally placed at the suprascapular area, shoulder, forearm, chest and abdomen were used. A correction factor was calculated to account for the evaporative resistance of the suit and compression top. To determine this correction factor, two post-hoc experiments were conducted. In both experiments, a water perfused mat (Gaymar T-Pad, Braintree Scientific Inc., MA, USA) was set to the average mean skin temperature under the top for the control (34.8 °C) or behavior (32.1 °C) trials. Four iButtons were set up to measure the absolute relative humidity at (1) Distance of 6mm above the mat under the suit and compression top, (2) A distance of 2 mm above the suit and compression top, (3) A distance of 6 mm above the mat outside of the suit and compression top and (4) A distance of ~10 mm above the mat (the same height as iButton 2, but without impedance from the suit and compression top). For the respective control or behavior trial, the suit top and a single layer of the compression top were placed over the iButtons. The suit top perfused 34 °C for the control trial, and 31.0° C for the behavior trial. A fully saturated paper towel was placed on top of the water perfused mat, underneath the iButton set up and the evaporation was measured for a 60 min period, as we have done previously [10]. The partial pressure of water at the mat (P_mat_) and at the garment (P_garment_) were calculated from Equation (14) below, averaging the first 20 min when the saturated paper towel mimicked the generation of sweat and a saturated skin. The correction factors (which were determined to be—Control: −1.58 kPa; Behavior: −1.18 kPa) were calculated as:

(14) $$P_{correction}=\;P_{mat}-\;P_{garment}\;(\mathrm{kPa})$$

H_evap_ under the suit was calculated as:

(15) $$E_{under}=h_{e}\;\times \;\left(Psk-\left(P_{a}-P_{correction}\right)\right)\times \;\mathrm{BSA}.\left(W\right)$$

Finally, respiratory heat losses were calculated for evaporation (E_res_) and convection (C_res_) as:

(16) $$\begin{array}{cc} E_{res}+\;C_{res}= & (0.0014\;\times \;\left(H_{prod}\right)\times \;\left(34-T_{a}\right)\\ & \;+(0.0173\;\times \;\left(H_{prod}\right)\times \;\left(5.87-P_{a}\right)\;\left(W\right) \end{array}$$
